# Phenylephrine Infusion Versus Bolus Regimens During Cesarean Delivery Under Spinal Anesthesia: An Observational Study to Assess Hemodynamic Changes

**DOI:** 10.7759/cureus.51977

**Published:** 2024-01-09

**Authors:** Harshni Kannan, Pughal Vendan Gnanaprakasam, Vishwanath Subramanian, Safneedha PM

**Affiliations:** 1 Department of Anesthesiology, Sri Muthukumaran Medical College Hospital and Research Institute, Chennai, IND

**Keywords:** cesarean delivery, phenylephrine bolus, phenylephrine infusion, regional anesthesia, maternal hypotension

## Abstract

Background

The objective of this study was to evaluate and compare the efficacy of two modes of phenylephrine administration, namely continuous infusion and intermittent bolus, in maintaining maternal hemodynamics during cesarean delivery under spinal anesthesia (SA).

Methods

Eighty patients undergoing cesarean delivery with SA were allocated into two groups. In group I, 40 patients were administered a prophylactic phenylephrine infusion at a rate of 75 mcg/min immediately after SA. Conversely, group B, consisting of 40 patients, received a 75 mcg bolus dose promptly after SA and subsequently whenever their blood pressure fell by more than 20% from the baseline value. Crucial variables, such as heart rate (HR), blood pressure, and side effects, were closely monitored at a three-minute interval in both groups. Following the delivery of the child, APGAR scores were documented at the first and fifth minutes, and the gathered data underwent analysis using SPSS Statistics, version 17.0 (SPSS Inc., Chicago, IL).

Results

The results revealed that baseline HR and blood pressure were similar in both groups. Nevertheless, the bolus group exhibited a higher mean HR, whereas the infusion group maintained a closer proximity to the baseline reading throughout the measurement period. Despite these variations, changes in HR did not demonstrate statistically significant differences between the two groups at any measuring intervals. Additionally, the mean systolic blood pressure in group B exhibited an initial decrease from the baseline, whereas group I displayed an increase compared to the baseline values. Importantly, neither group reported instances of nausea or vomiting, and the APGAR scores were comparable between them.

Conclusion

In conclusion, the study found that a phenylephrine bolus of 75 mcg was more effective in maintaining blood pressure within acceptable limits without causing bradycardia or hypertension when compared to a phenylephrine infusion.

## Introduction

Cesarean sections represent one of the most commonly performed surgical procedures, often conducted under regional anesthesia due to its numerous advantages. Regional anesthesia offers rapid onset, the simultaneous blockade of sensory and motor pathways, coupled with a decreased risk of local anesthetic drug toxicity, establishing it as the preferred technique in this clinical context [1].

However, a notable and undesirable consequence associated with the subarachnoid block, a key component of regional anesthesia in cesarean sections, is a significant drop in blood pressure. This phenomenon, known as hypotension, is a well-documented concern as it can potentially harm both the mother and the baby [2,3]. Various preventive measures have been explored to mitigate this issue, including preloading the patient with intravenous fluids [4], reducing the dose of subarachnoid block [5], and employing various drug regimens aimed at increasing blood pressure [6]. Despite the effectiveness of these preventive strategies, it remains essential to continuously monitor blood pressure during the procedure to promptly address maternal hypotension, as underscored by a comprehensive Cochrane review [7].

Maternal hypotension resulting from subarachnoid blockade can give rise to a range of maternal and fetal side effects. Mothers experiencing hypotension often exhibit symptoms such as vomiting, nausea, and a feeling of impending distress, which can be attributed to reduced blood circulation to the brain. The fetus is indirectly affected by the drop in maternal blood pressure, as it relies on the pressure within the maternal uterine artery to maintain adequate uterine blood flow. Without timely intervention, fetal acidosis, a condition detrimental to the unborn child, may ensue [3].

Ephedrine has been the preferred medication for addressing maternal hypotension in obstetric settings [8]. Nevertheless, recent research [9] has prompted concerns about its potential impact on fetal pH, without a concurrent alteration in the APGAR score, which could potentially result in fetal acidosis. In more recent times, phenylephrine has emerged as the favored vasopressor for preventing and managing maternal hypotension during cesarean sections. This preference is attributed to its lower incidence of fetal acidosis compared to ephedrine [8-11].

Phenylephrine, particularly as an alpha agonist, has gained endorsement for treating the post-spinal anesthesia (SA) fall in blood pressure in obstetric contexts due to its superior safety profile regarding fetal acidosis when compared to ephedrine [9-11].

While the current practice includes the administration of phenylephrine through both continuous infusion and intermittent boluses, the ideal dosage strategy remains undefined. These two distinct techniques have been studied for their respective effects on baroreceptor sensitivity [12-15], but direct comparisons are lacking, especially concerning their ability to manage maternal blood pressure close to standard values throughout cesarean sections conducted under SA. This gap in the literature highlights the need for a comprehensive investigation, which forms the basis of the current study.

## Materials and methods

Study participants and group allocation

This study encompassed 80 patients undergoing cesarean delivery with SA, divided into two groups. In group I, 40 patients were administered a prophylactic phenylephrine infusion at a rate of 75 mcg/min immediately after SA. Meanwhile, group B, comprising 40 patients, received a 75 mcg bolus dose of phenylephrine right after SA and additionally whenever their blood pressure dropped by more than 20% from the baseline value.

Type of study

Prospective Observational Study

The study design is prospective as it follows patients forward in time from the point of receiving SA during cesarean delivery.

Interventional Components

Although observational, the study involves specific interventions in terms of administering phenylephrine to the participants in two different ways.

Focus on Cesarean Delivery

The study is specifically focused on patients undergoing cesarean delivery with SA.

Inclusion criteria

Patients Undergoing Cesarean Delivery

Only those patients who were opting for cesarean delivery were included in the study.

SA Recipients

Participants were those receiving SA for the procedure.

Consent to Participate

Patients who provided written informed consent were included.

Ethics Committee Approval

Participants were included after formal clearance from the institutional ethics committee.

Exclusion criteria

(Not explicitly stated in the provided text, but generally assumed based on standard clinical study practices)

Non-consenting Patients

Those who did not provide informed consent would be excluded.

Contraindications to Study Procedures

Potential participants with contraindications to SA, phenylephrine, or any aspect of the study protocol would likely be excluded.

Patients Not Opting for Cesarean Delivery

As the focus is on cesarean delivery, patients opting for vaginal delivery would be excluded.

Severe Preexisting Conditions

Patients with severe preexisting medical conditions that could interfere with the study outcomes (e.g., severe cardiovascular diseases and allergic reactions to study medications) might be excluded.

Data collection

Several outcome variables, such as heart rate (HR), blood pressure, and the occurrence of side effects, were meticulously documented for each participant in the study. Furthermore, the APGAR score was evaluated at both the first and fifth minutes after the child’s delivery. Following this, the gathered data underwent thorough analysis.

Patient recruitment and informed consent

Participants were recruited through convenience sampling, and all individuals provided written informed consent to take part in this observational study following formal approval from the institutional ethics committee. A thorough pre-anesthetic assessment was conducted for each patient, during which they were informed about the subarachnoid block procedure and the potential benefits and risks associated with the use of phenylephrine.

Preoperative preparation and monitoring and anesthetic procedure

In the operating theater, standard monitoring procedures were executed, encompassing mean arterial pressure, oxygen saturation, HR, and blood pressure measurements. Intravenous access was established, and baseline readings for these parameters were meticulously recorded.

Patients were placed in a left lateral position, and a skilled anesthesiologist administered SA using 9-11 mg of 0.5% hyperbaric bupivacaine. Following the subarachnoid block, patients were repositioned in a supine position with a wedge beneath the right flank.

Throughout the study duration, starting from the administration of SA, parameters such as oxygen saturation, blood pressure, and HR were documented at a three-minute interval. A 60cc syringe containing a solution with 25 mcg of phenylephrine per cc was used. In group I, patients received an initial 75 mcg (equivalent to 3 cc) per minute of phenylephrine infusion immediately after SA. The infusion continued for the initial three minutes, unless the blood pressure exceeded 20% of the baseline value. Adjustments to the infusion rate were based on blood pressure readings. If the blood pressure remained at or below the baseline, the infusion was maintained. In group B, patients received an immediate prophylactic phenylephrine bolus of 75 mcg following SA, with intermittent boluses of 75 mcg administered whenever there was a decline in blood pressure. Hypotension was defined as a drop of more than 20% from baseline in systolic or diastolic blood pressure (DBP), and reactive hypertension was noted when the blood pressure exceeded 20% of the baseline value. A specific protocol was followed for managing bradycardia, defined as a HR dropping below 60 beats per minute, during cesarean section under SA. If bradycardia occurred, the phenylephrine infusion was immediately stopped, and a dose of 0.6 mg of atropine was administered as a corrective measure. Additionally, to assess neonatal well-being, the APGAR scores were meticulously recorded at one and five minutes post-delivery. This protocol ensured both maternal safety in response to hemodynamic changes and the monitoring of neonatal health immediately after birth.

Statistical analysis

The statistical analysis was done using SPSS Statistics, version 17.0 (SPSS Inc., Chicago, IL). The methodology encompassed a comprehensive examination of demographic characteristics and baseline parameters among the 80 patients undergoing cesarean delivery with SA, divided into groups I and B. The demographic variables, including age, weight, and height, were meticulously scrutinized using SPSS to calculate mean and standard deviation, with subsequent application of independent t-tests to discern any statistically significant differences between the two groups. Subsequently, the study focused on monitoring HR, systolic blood pressure (SBP), and DBP at specific intervals (zero, three, six, and nine minutes) using SPSS for mean calculation. Independent t-tests were then employed to assess the statistical significance of mean differences between groups I and B at these time points. Furthermore, SPSS facilitated an in-depth examination of HR changes at three, six, and nine minutes post-SA, revealing no statistically significant differences between the two groups. The analysis extended to blood pressure changes, highlighting significant differences in SBP at three and nine minutes, favoring bolus administration, as well as DBP at three minutes. Fisher’s exact test was applied to categorical data on blood pressure changes. Finally, the well-being of newborns was evaluated through APGAR scores at one and five minutes, with SPSS and independent t-tests employed to determine if any significant differences existed between groups I and B in this regard. This rigorous statistical approach provided valuable insights into the comparative effects of phenylephrine administration methods during cesarean delivery.

## Results

**Figure 1:**
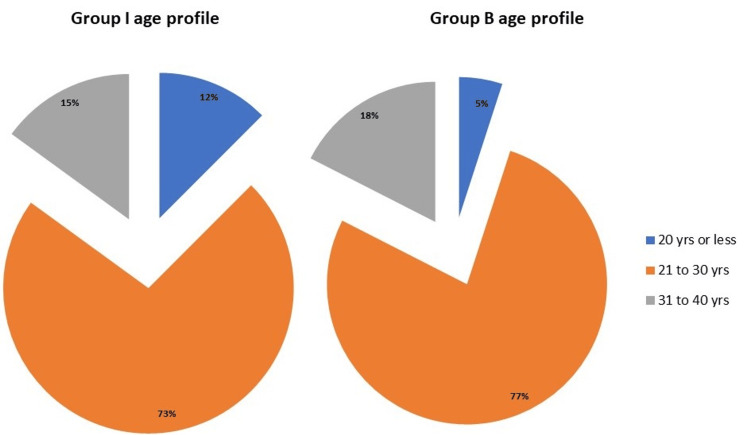
Age profiles of patients in groups I and B

Eighty patients undergoing cesarean delivery with SA participated in the study, and they were categorized into two groups: groups I and B. Importantly, the demographic characteristics of the participants were carefully examined. These included their heights, weights, and ages. Specifically, the age distribution showed that the majority of patients in both groups fell within the 21-30 years age group (group I: 73%; group B: 77%) (Figure 1). 

As shown in Table 1, the mean patient weights in groups I and B were 58.63 and 60.13 kg, respectively, and no significant differences in weight were seen among both groups (p = 0.291). The mean patient heights in groups I and B were 158.5 and 158.7 cm, respectively, with no appreciable difference among both groups (p = 0.87).

**Table 1:** Group-wise demographic and physiological profiles of patients HR, heart rate; SBP, systolic blood pressure; DBP, diastolic blood pressure

Group	N	Mean age group	Mean weight (kg)/std. dev	Mean height (cm)/std. dev	HR	SBP	DBP
Group I	40	24.38	58.63 ± 6.72	158.5 ± 5.85	75.78	116.4	70.9
Group B	40	27.68	60.13 ± 5.86	158.7 ± 5.01	71.95	116.2	72.35
Total	80		59.38 ± 6.31	158.6 ± 5.41			

The study assessed various baseline parameters, including HR, SBP, and DBP, to establish a baseline for comparison. The results demonstrated no significant differences in baseline HR between the two groups (p = 0.126).

**Figure 2:**
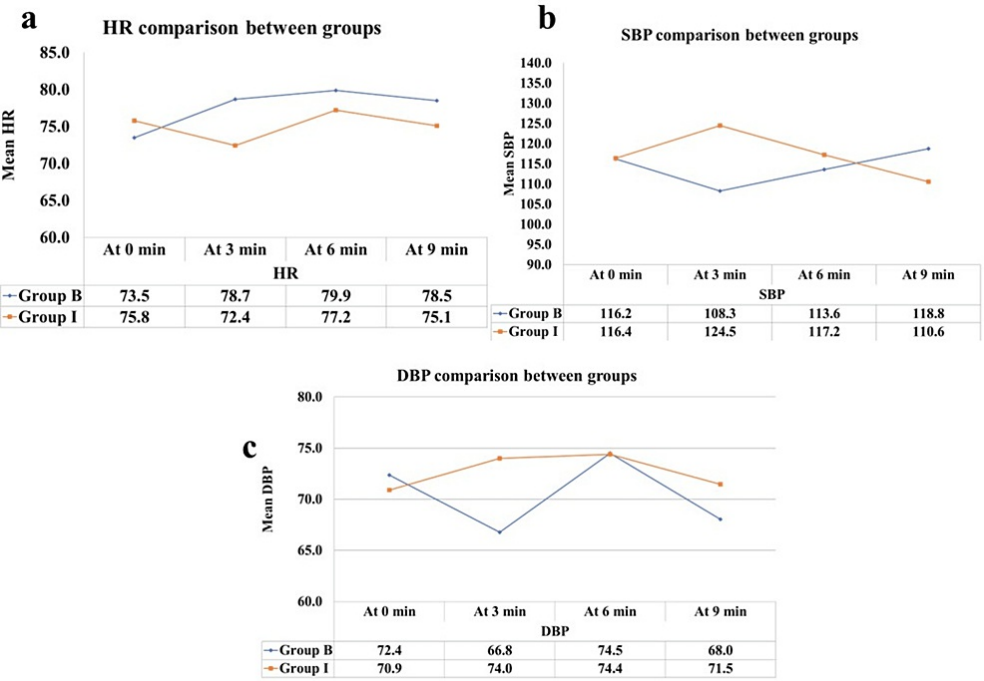
Comparative analysis of cardiovascular parameters between groups HR, heart rate; SBP, systolic blood pressure; DBP, diastolic blood pressure

The study monitored HR throughout the procedure and compared it between the two groups. While the baseline HR was similar between the groups, significant differences emerged when evaluating the mean HR at three, six, and nine minutes following SA. Notably, the bolus group exhibited a higher mean HR than the infusion group at these time points, as evidenced in Figure 2. However, Fisher’s exact test and t-test revealed that the changes in HR were not significant from a statistical viewpoint between the two groups at any measuring intervals (p > 0.072). Table 2 presents the distribution of increased, decreased, and <20% HR changes in groups B and I. Notably, group B exhibited a higher count of increased HRs (>20%) compared to group I (30.0% vs. 2.5%). Conversely, a decrease in HR >20% is a bad event; we looked at incidents of such an event in two groups, and it’s significantly higher in group I. The majority of cases in both groups had HR changes <20% (67.5% in group B and 77.5% in group I).

**Table 2:** Heart rate (HR) parameters: distribution of increased and decreased rates (>20%) in groups B and I patients Fisher’s exact test; p = 0.001, HS

	Increased (>20%)	Heart rate between the groups			
		B		I	
		Count	Column, N%	Count	Column, N%
		12	30.0%	1	2.5%
	Decreased (>20%)	1	2.5%	8	20.0%
	<20%	27	67.5%	31	77.5%
	Total	40	100.0%	40	100.0%

Table 3 provides a detailed comparison of HRs between groups B and I at different time intervals (zero, three, six, and nine minutes). At each time point, the mean HRs were calculated along with standard deviation and 95% confidence intervals. The t-test p-values indicate the statistical significance of the differences in HRs between the two groups. Notably, no statistically significant differences were observed at any time point (p > 0.05). Blood pressure, particularly SBP, was another crucial parameter that was examined closely. Table 4 outlines the distribution of increased, decreased, and <20% SBP changes in groups B and I. Group B showed no instances of increased SBP (>20%), while group I had a notable count (12.5%). On the contrary, group B had a higher count of decreased SBP (>20%) compared to group I (5.0% vs. 0.0%). The majority of cases in both groups had SBP changes <20% (95.0% in group B and 87.5% in group I). The study found that the mean baseline SBP was similar among both groups (p = 0.940). However, distinctive trends in SBP were observed. Group B exhibited a significant drop from the baseline at the three-minute interval following SA, while group I showed a notable rise compared to the baseline value, as evidenced in Table 4 and Figure 2. These differences were statistically significant (p = 0.000).

**Table 3:** Comparative analysis of heart rate (HR) between groups at different time intervals: mean, standard deviation, 95% confidence intervals, and t-test results

Time	Group	N	Mean	Std. deviation	95% confidence interval for mean		t-test p-value	
					Lower bound	Upper bound		
At 0 min	B	40	73.45	10.21	70.19	76.71	0.342	NS
	I	40	75.78	11.51	72.09	79.46		
At 3 min	B	40	78.68	12.46	74.69	82.66	0.072	NS
	I	40	72.40	17.84	66.70	78.10		
At 6 min	B	40	79.88	12.09	76.01	83.74	0.415	NS
	I	40	77.18	16.97	71.75	82.60		
At 9 min	B	40	78.48	13.50	74.16	82.79	0.268	NS
	I	40	75.08	13.74	70.68	79.47		

**Table 4:** Systolic blood pressure (SBP) parameters: distribution of increased and decreased rates (>20%) in groups B and I patients Fisher’s exact test; p = 0.024, sig

Increased (>20%)	Group			
	B		I	
	Count	Column, N %	Count	Column, N %
	0	0%	5	12.5%
Decreased (>20%)	2	5.0%	0	0.0%
<20%	38	95.0%	35	87.5%
Total	40	100.0%	40	100.0%

The data revealed that phenylephrine bolus administration was more effective at maintaining blood pressure within the acceptable limits (±20% from baseline) when compared to phenylephrine infusion. Moreover, it achieved this without inducing undesirable side effects such as reflex bradycardia and reactive hypertension, which were observed more frequently in group I. Table 5 presents a comparative analysis of SBP between groups B and I at different time intervals (zero, three, six, and nine minutes). The mean SBP, standard deviation, and 95% confidence intervals are reported, along with t-test p-values indicating the statistical significance of the differences. Statistically significant differences in SBP were observed at the three- and nine-minute marks.

**Table 5:** Comparative analysis of systolic blood pressure (SBP) between groups at different time intervals

Time	Group	N	Mean	Std. deviation	95% confidence interval for mean		t-test p-value	
					Lower bound	Upper bound		
At 0 min	B	40	116.20	11.22	112.61	119.79	0.940	NS
	I	40	116.40	12.35	112.45	120.35		
At 3 min	B	40	108.25	12.56	104.23	112.27	0.000	HS
	I	40	124.50	24.25	116.74	132.26		
At 6 min	B	40	113.58	10.68	110.16	116.99	0.332	NS
	I	40	117.23	21.12	110.47	123.98		
At 9 min	B	40	118.75	8.10	116.16	121.34	0.020	Sig
	I	40	110.60	20.21	104.14	117.06		

Table 6 details the comparative analysis of DBP between groups B and I at various time intervals (zero, three, six, and nine minutes). The mean DBP, standard deviation, and 95% confidence intervals are presented, along with t-test p-values indicating the statistical significance of the differences. Notably, statistically significant differences in DBP were observed at the three-minute mark. There was a higher incidence of hypertension and bradycardia in group I when compared to group B. This could be attributed to the higher dosage of phenylephrine administered in infusion when compared to bolus doses.

**Table 6:** Comparative analysis of diastolic blood pressure (DBP) between groups at different time intervals

Time	Group	N	Mean	Std. deviation	95% confidence interval for mean		t-test p-value	
					Lower bound	Upper bound		
At 0 min	B	40	72.35	10.47	69.00	75.70	0.515	NS
	I	40	70.90	9.32	67.92	73.88		
At 3 min	B	40	66.78	7.20	64.47	69.08	0.004	HS
	I	40	74.00	13.55	69.67	78.33		
At 6 min	B	40	74.50	8.92	71.65	77.35	0.964	NS
	I	40	74.38	15.03	69.57	79.18		
At 9 min	B	40	68.03	5.39	66.30	69.75	0.138	NS
	I	40	71.48	13.53	67.15	75.80		

The study also assessed the well-being of the newborns by comparing APGAR scores at one and five minutes after birth between the two groups. The data showed no statistically significant differences between the two groups in terms of APGAR scores, as demonstrated in Table 7. Furthermore, neither group reported any episodes of nausea or vomiting.

**Table 7:** Baby’s APGAR score at one and five minutes after birth

Group	Mean and std. dev		t-test p-value	
	1 min	5 min	1 min	5 min
B	8.175 ± 0.84	9.87 ± 0.33	0.6973	0.3718
I	8.25 ± 0.86	9.8 ± 0.40		

## Discussion

The study primarily investigates how different administration methods of phenylephrine influence maternal hemodynamics during cesarean sections under SA. This includes examining the differential impact on HR and systemic vascular resistance, which are crucial for ensuring maternal safety and fetal well-being. Understanding the hemodynamic changes, especially the baroreceptor-mediated bradycardia, in response to phenylephrine administered via different modes is central to optimizing patient care during cesarean sections. This approach is pivotal in enhancing our understanding of the most effective and safest administration practices for phenylephrine in this clinical setting.

Recent research showed comparison of an infusion group receiving 50 mcg/min of phenylephrine with a bolus group. In their study, the phenylephrine dosage was considerably lower than that in our current investigation. Nevertheless, our study revealed that the prophylactic bolus administration of 75 mcg of phenylephrine resulted in hypotension in approximately 5% of patients. Despite these hypotensive episodes, the three-minute SBP trend following SA demonstrated effective control of maternal SBP, staying within 20% of the baseline, without reporting hypertension in any study participants. Furthermore, maternal HR was maintained within 20% of the baseline, with only 2.5% of patients experiencing bradycardia [16].

In our study, our primary goal was to sustain maternal HR and blood pressure close to baseline levels, acknowledging the critical importance of maternal hemodynamics in ensuring sufficient uteroplacental blood flow. The context of cesarean sections presents a distinctive challenge, as central neuraxial blockade frequently leads to a sudden decrease in maternal blood pressure. The enlarged uterus, compressing the inferior vena cava and aorta, adds complexity to this situation. This compression hampers venous return and, consequently, maternal cardiac output. Any substantial decline in maternal blood pressure or HR can have profound implications for fetal well-being.

Lakshmikanthan et al. discussed a study that emphasized the limited success of preloading patients with crystalloids, with 85% of the study population still experiencing hypotension [1]. The authors underscored the importance of selecting an ideal vasopressor, as it significantly impacts cardiac output, maternal HR, and SBP, ultimately influencing fetal outcomes. Their study achieved better results with the use of phenylephrine infusion [1].

In our current study, while the infusion group exhibited better control over SBP, with no cases of hypotension, an interesting finding emerged. The three-minute SBP trend following SA showed an increase in SBP of more than 20% from baseline, indicating episodes of hypertension in 12.5% of group I. Intriguingly, despite maintaining SBP within acceptable limits, the infusion group failed to maintain HR, with 20% of patients experiencing bradycardia, another significant side effect of phenylephrine.

Dyer et al. conducted a study exploring the effects of bolus doses of phenylephrine on maternal HR and SBP [17]. Their findings concluded that small rescue boluses of phenylephrine were effective in maintaining SBP near baseline levels. The key to monitoring cardiac output in their study was HR [17].

Similarly, Lee et al. performed a study involving prophylactic bolus doses of phenylephrine. They reported hypotension in almost 37% of the study population who received a bolus dose of 1.5 mcg/kg of phenylephrine, necessitating the use of 100 mcg phenylephrine rescue doses [18].

A major limitation observed in previous studies was the challenge of selecting the optimal phenylephrine dosage and administration method to maintain maternal vital signs without adverse effects while promoting favorable fetal outcomes [19].

In research conducted by Dusitkasem in 2001, they explored the impact of prophylactic phenylephrine infusion on 75 women undergoing cesarean delivery following SA [8]. Their findings revealed improved fetal outcomes, indicated by a higher pH in blood gas analysis, and a decrease in adverse effects such as nausea or vomiting when maternal SBP was closely regulated. Significantly, our current study observed no occurrences of nausea and vomiting within the study population. Favorable fetal outcomes were also noted in both groups, as demonstrated by APGAR scores.

Overall, our study provides an understanding of phenylephrine administration in the context of maternal hemodynamics during cesarean sections. The choice between prophylactic infusion and bolus administration should consider not only the control of SBP but also the overall maternal and fetal well-being [19].

## Conclusions

In conclusion, the study explores optimal phenylephrine administration to maintain maternal SBP during cesarean sections with SA. Prophylactic phenylephrine infusion at 75 mcg/min achieved tighter SBP control but led to undesired effects, including reactive hypertension (12.5%) and bradycardia (20%). In contrast, prophylactic bolus administration of 75 mcg regulated SBP within acceptable limits (±20% from baseline) without adverse events. Although 5% experienced hypotension, it was promptly managed with a phenylephrine rescue dose. APGAR scores at one and five minutes were comparable between groups. Notably, PG1 suggests it as a preferable approach for effective SBP control with fewer adverse effects.
